# Prevalence of Depression Among Students of Urmia University of Medical Sciences (Iran)

**Published:** 2011

**Authors:** Nader Aghakhani, Hamid Sharif Nia, Samereh Eghtedar, Narges Rahbar, Madineh Jasemi, Maryam Mesgar Zadeh

**Affiliations:** 1 MSc,* Candidate of PhD in Nursing*, Department of Medical Surgical Nursing, School of Nursing and Midwifery, Urmia University of Medical Sciences, Urmia, Iran.; 2 MSc*, Candidate of PhD in Nursing*, Department of Medical Surgical Nursing, School of Nursing and Midwifery, Babol University of Medical Sciences, Babol, Iran.; 3 MSc, Department of Medical Surgical Nursing, School of Nursing and Midwifery, Urmia University of Medical Sciences, Urmia, Iran.

**Keywords:** Depression, Medical Students, Prevalence, Urmia

## Abstract

**Objective:** A depressive disorder is an illness that involves the body, mood, thoughts and behaviors. This study was performed to identify the presence of depression among medical students of Urmia University of Medical Sciences.

**Methods:** A descriptive cross-sectional study was conducted on 700 undergraduate medical and basic sciences students. Beck depression inventory (BDI) used for data gathering.

**Results:** Mean score of BDI was 10.4 **± **0.8 and 52.6% of students scored under the depression threshold. Four of them had severe depression. Results showed no significant relationship between depression and age, education, sex, rank of birth or duration of education.

**Conclusion:** Prevalence of depression that can affect the students’ quality of education and social behavior was high in Urmia University of Medical Sciences.

## Introduction

Due to its prevalence and associated consequences, depression is an important health problem. With a worldwide prevalence of about 10-15%, it is one of the most frequent and debilitating mental disorders (1). According to the precipitation of the World Health Organization (WHO), depression is estimated to become the second leading cause of dysfunction by the year 2020 (2). It is well known that stressful life events can cause psychological symptoms. Besides, stressful life events have been suggested to be antecedents and even predictors of the majority of depression symptoms (3).

Depression as a mood disorder is one of the most basic problems of human health. It is a very serious difficulty which costs a lot for the countries. Depression is increasingly viewed as a chronic illness (4), according to the fact that depressed individuals experience high rates of symptom recurrence (5) and sustained functional impairment (6). In recognition of the chronic condition, most trials of depression treatment incorporate principles of chronic disease management into the interventions tested (7).

A depressive disorder is an illness that involves the body, mood, thoughts and behaviors. It affects the way a person eats, feels, sleeps and thinks about things (8). Depressed individuals may suicide without a specific plan (9). The economic cost of this disorder is high; however, the costs of human suffering cannot be estimated (10). Adolescents with sub-diagnostic levels of depressive symptoms show higher rates of early-adulthood depression, substance misuse, adverse psychological and social functioning. When severity of symptoms reaches the diagnostic threshold, depression will likely continue until early adult life (11).

Because of its recurrence and complications, this problem is very important, especially in the youth and students of universities. Studies have shown that depression in young people is a risk factor for suicide, increased risk-taking behaviors (e.g. substance abuse, early onset sexual experiences), teenage pregnancy, adulthood depression, conduct disorder and delinquency (12). Suicide due to depression is one of the major causes of adolescent’s mortality in developed countries (13).

Published studies from Iran have shown that the prevalence of depression amongst students of universities varies from 36% to 66%, about 2-5% of whom suffer from a severe form of disorder (14).

Multiple risk factors may increase the risk of depression among university students such as being away from home, experiencing reduced adult supervision and family problems (15,16). According to the published studies, university students from all over the world are vulnerable to serious mental health problems especially depression, anxiety and suicide (17,18).

The present study was performed to determine the prevalence of depression among students of Urmia University of Medical Sciences, Iran.

## Methods and Materials

Urmia, which is located in north-west of Iran, has the population of 3200000. Urmia University of Medical Sciences had 700 medical students at time of this research. Permission for the study was granted by ethics committee of Urmia medical university. Verbal consent was obtained from participants in classrooms and only the consenting students filled the anonymous questionnaire.

This study was performed as a cross-sectional study in Urmia University of Medical Sciences and a questionnaire evaluating depression was distributed to students from different specialties. Students were a random sample of all students of the university.

Data were collected through Beck depression inventory (BDI): A 21-item questionnaire presented in multiple-choice format to measure presence and degree of depression in adolescents and adults.

Each of the items of the BDI attempts to assess a specific symptom or attitude, which appears to be specific to depressed patients and is in consistent with descriptions of the depression contained in the psychiatric literature.

This questionnaire consists of 21 multiple-choice questions scored by simple Likert method [0, 1, 2, 3]. Minimum score is 0 and maximum is 63. The range of 0-9 is considered as the minimum, 10-16 as the minor mood disturbance, 17-29 as the average clinical depression and 30-63 as the severe depression. We considered a group (≥ 40) by ourselves for recognizing higher levels of depression.

BDI studies disappointment feeling of defeat, hopelessness, feelings of guilt, worthlessness, dissatisfaction, sinfulness, being punished, being disgusted, being reproached, inclination to suicide weeping, being touchy, apathy to people, inability for making a decision, bad feeling of appearance, insomnia, fatigue, loss of appetite, loss of weight, anxiety about health and decrease in sexual activities, loss of interest or pleasure in hobbies and decreased energy.

Validity and reliability of Farsi version of BDI have been demonstrated in Iran (3).

A single paged questionnaire intended to obtain demographic information of the study population [i.e. age, gender, marital status (single/married), being native (yes/no), parents (alive/death), rank of birth and duration of education].

The self-administered questionnaires were distributed among 700 medical students, 628 (89.7%) of whom completed the questionnaire.

Anonymity of participants and confidentiality of information were assured. All collected data were considered confidential and were handled only by the investigators. The analysis was performed using SPSS 16.0. Descriptive and inferential statistics were performed for the dependent and independent variables respectively. A probability level of < 0.05 was considered statistically significant.

## Results

This study included 628 participants, 53.2% of whom were male and 84% were single. The age ranged from 18 to 26 years with a mean age of 22 **±** 0.3 years. About 52.6% suffered from different degrees of depression and severe depression was observed in 4% (CI95: 2.47-5.52).

The number of filled returned BDI questionnaires was 628. From all participants, 298 (47.4%) had a BDI score in the minimum range (0-9), 197 (31.3%) in the minor mood disturbance range (10-16), 91 (14.5%) in the depression range (17-29), and 28 (4.5%) had their BDI score in the severe depression range (30-63). Only 12 (2%) had a BDI score of ≥40 indicating severe depression. The mean score of depression was 10.4. Based on BID, 52.6% were depressed at different degrees.

The difference of depression score was not statistically significant between males and females (p=0.08) and married and singles (p=0.06). Loss of father did not affect the depression degree (p=0.06) significantly, whereas the effect of loss of mother was statistically significant (p<0.001).

Its severity was higher in the students whose mothers died and in non–native students (p=0.02) as well. Its frequency had no significant relation with age (p=0.04), rank of birth (p=0.05) and duration of education (p=0.06), whereas there was a significant relation between depression and the bad marks in their lessons (p=0.05). 36.4% of respondents were not native. Father of 13.7% and mother of 3.5% of them were dead.

Nursing students were more depressed than others. Being native or not had no effect on depression (p=0.1). Its level had no relation with age (p=0.06), rank of birth (p=0.1) and duration of education (p=0.08), whereas it had a significant relation with situation of education and bad marks in lessons (p=0.04). 

## Discussion

Depression is caused by a chemical imbalance in the central nervous system. Although the exact mechanism is unknown, bouts of depression can be triggered by sad or stressful life events, hormonal changes, disease or certain medications. People with a low self-esteem and a pessimistic outlook on life seem to be particularly prone to depression (1).

Level of depression, which can affect the quality of education and social behavior of the students, was very high in Urmia University. Its reasons can be unemployment, economic inability to get married and to continue education. About 30% of students are depressed at the beginning of university education and the rate of depression increases gradually.

In Malaysia, 41.9% of medical students were found to have emotional disturbances. 47.9% of second year medical students in Antalya, Turkey, were found to have emotional disorders, higher than the percentage of students studying economics (29.2%) and physical education (29.2%) (7).

Lotfi et al (2010) indicated that 50% of Yazd (Iran) students were suffering from different grades of depression, 35.4% of which was mild, 13.4% was moderate, and only 1.2% was severe (14). It was identified in a study performed in Ardebil University of Medical Sciences (Iran) that 57.6% of students were suffering from depression, 39.8% of which was mild, 14.8% was moderate and 3% was severe (19).

Students with high intelligence, good problem-solving and social skills, high self-esteem, and sense of control and positive expectations of the future are less likely to become depressed at the presence of environmental risk factors. Depression is present among nursing students at the same levels as expected for the non–diagnosed population. Depression is associated with medium levels of self–esteem. In addition to the characterized emotional state of depression, depressed students notice physical health problems, although they do not associate them with this condition (20).

A positive attribution style provides protection against stressful life events. In addition to such individual characteristics, the presence of social support plays an important protective role. Such support includes good peer relations, support from teachers and a stable relationship with at least one parent (21).

The best thing that can be done is to help them be treated by encouraging them to seek professional helps and offering emotional support. They must not be given negative thinking. On the other hand, they must take an active role in getting better and must be good to themselves, when they are getting well. Internal changes are needed in problem assessment, self-evaluation and the expectations of himself / herself. External changes like recreations, stress management, communication and medication can be used in relieving the symptoms and can make significant improvement in patients’ mood and life adjustment. More researches must be done about treatments .It is important to investigate the causes of depression and plan to omit them.

Special attention should be paid to the changes in the depressive state among students, and their search for frequent clinical services and extra–class activities in the field should be observed (19). Establishment of consulting and recreation centers can be helpful.

## Authors’ Contributions

NA conceived and designed the evaluation, supervised the process of research and helped to draft the manuscript, HS participated in designing the evaluation and performed parts of the statistical analysis and coordinate the members of research. SE collected clinical data, interpreted them, performed the statistical analysis and revised the manuscript. NR, MJ and MMZ collected some parts of clinical data, re-evaluated them, re-analyzed the statistical data and revised the manuscript. All authors read and approved the final manuscript.

## Notes

**Declaration of Interest:** None.
